# Lessons Learned during Public Health Response to Cholera Epidemic in Haiti and the Dominican Republic

**DOI:** 10.3201/eid1711.110827

**Published:** 2011-11

**Authors:** Jordan W. Tappero, Robert V. Tauxe

**Affiliations:** Centers for Disease Control and Prevention, Atlanta, Georgia, USA

**Keywords:** Cholera, Haiti, Dominican Republic, waterborne disease, public health response, epidemiology, sanitation, epidemic control, bacteria, safe water, sewage, infrastructure

## Abstract

After epidemic cholera emerged in Haiti in October 2010, the disease spread rapidly in a country devastated by an earthquake earlier that year, in a population with a high proportion of infant deaths, poor nutrition, and frequent infectious diseases such as HIV infection, tuberculosis, and malaria. Many nations, multinational agencies, and nongovernmental organizations rapidly mobilized to assist Haiti. The US government provided emergency response through the Office of Foreign Disaster Assistance of the US Agency for International Development and the Centers for Disease Control and Prevention. This report summarizes the participation by the Centers and its partners. The efforts needed to reduce the spread of the epidemic and prevent deaths highlight the need for safe drinking water and basic medical care in such difficult circumstances and the need for rebuilding water, sanitation, and public health systems to prevent future epidemics.

## Abstract 2

Safe water and sewage systems must be constructed to prevent future epidemics.

Cholera is a severe intestinal infection caused by strains of the bacteria *Vibrio cholerae* serogroup O1 or O139, which produce cholera toxin. Symptoms and signs can range from asymptomatic carriage to severe diarrhea, vomiting, and profound shock. Untreated cholera is fatal in ≈25% of cases, but with aggressive volume and electrolyte replacement, the number of persons who die of cholera is limited to <1%. Since 1817, cholera has spread throughout the world in 7 major pandemic waves; the current and longest pandemic started in 1961 (*1*). This seventh pandemic, caused by the El Tor biotype of *V. cholerae* O1 and O139, began in Indonesia, spread through Asia, and reached Africa in 1971. In 1991, it appeared unexpectedly in Latin America, causing 1 million reported cases and 9,170 deaths in the first 3 years (*2*). The other biotype of *V. cholerae* O1, called the classical biotype, is now rarely seen.

Cholera is transmitted by water or food that has been contaminated with infective feces. The risk for transmission can be greatly reduced by disinfecting drinking water, separating human sewage from water supplies, and preventing food contamination. Industrialized countries have not experienced epidemic cholera since the late 1800s because of their water and sanitation systems (*3*). The risk for sustained epidemics may be associated with the infant mortality rate (IMR) because many diarrheal illnesses of infants spread through the same route. In Latin America, sustained cholera transmission was seen only in countries with a national IMR >40 per 1,000 live births (*4*). Although cholera persists in Africa and southern Asia, it recently disappeared from Latin America after sustained improvements in sanitation and water purification (*5*,*6*). Although the country was at risk, until the recent outbreak, epidemic cholera had not been reported in Haiti since the 1800s, and Haiti, like other Caribbean nations, was unaffected during the Latin America epidemic (*7*,*8*).

## Haiti: A History of Poverty and Poor Health

Haiti has extremely poor health indices. The life expectancy at birth is 61 years (*9*), and the estimated IMR is 64 per 1,000 live births, the highest in the Western Hemisphere. An estimated 87 of every 1,000 children born die by the age of 5 years (*9*), and >25% of surviving children experience chronic undernutrition or stunted growth (*10*). Maternal mortality rate is 630 per 100,000 live births (*10*).

Haitians are at risk of spreading vaccine-preventable diseases, such as polio and measles, because childhood vaccination coverage is low (59%) for polio, measles-rubella, and diphtheria-tetanus-pertussis vaccines (*9*). Prevalence of adult HIV infection (1.9%) and tuberculosis (312 cases per 100,000 population) in the Western Hemisphere is also highest in Haiti (*11*,*12*), and Hispaniola, which Haiti shares with the Dominican Republic, is the only Caribbean island where malaria remains endemic (*13*).

Only half of the Haitian population has access to health care because of poverty and a shortage of health care professionals (1 physician and 1.8 nurses per 10,000 population), and only one fourth of seriously ill persons are taken to a health facility (*14*). Before the earthquake hit Haiti in January 2010, only 63% of Haiti’s population had access to an improved drinking water source (e.g., water from a well or pipe), and only 17% had access to a latrine (*15*).

## Aftermath of Earthquake

The earthquake of January 12, 2010, destroyed homes, schools, government buildings, and roads around Port-au-Prince; it killed 230,000 persons and injured 300,000. Two million residents sought temporary shelter, many in internally displaced person (IDP) camps, while an estimated 600,000 persons moved to undamaged locations.

In response, the Haitian government developed strategies for health reform and earthquake response (*16*,*17*) and called on the international community for assistance. The Ministère de la Santé Publique et de la Population (MSPP) requested assistance from the Centers for Disease Control and Prevention (CDC) to strengthen reportable disease surveillance at 51 health facilities that were conducting monitoring and evaluation with support from the US President’s Emergency Plan for AIDS Relief (PEPFAR) (*18*) and at health clinics for IDPs (*19*). MSPP also asked CDC to help expand capacity at the Haiti Laboratoire National de Sante Publique to identify reportable pathogens, including *V. cholerae* (*20*,*21*), and help train Haiti’s future epidemiologic and laboratory workforce. These actions, supported through new emergency US government (USG) funds to assist Haiti after the earthquake, laid the groundwork for the rapid detection of cholera when it appeared.

## Cholera Outbreak

On October 19, 2010, MSPP was notified of a sudden increase in patients with acute watery diarrhea and dehydration in the Artibonite and Plateau Centrale Departments. The Laboratoire National de Sante Publique tested stool cultures collected that same day and confirmed *V. cholerae* serogroup O1, biotype Ogawa, on October 21. The outbreak was publicly announced on October 22 (*22*).

A joint MSPP-CDC investigation team visited 5 hospitals and interviewed 27 patients who resided in communities along the Artibonite River or who worked in nearby rice fields (*23*). Many patients said they drank untreated river water before they became ill, and few had defecated in a latrine. Health authorities quickly advised community members to boil or chlorinate their drinking water and to bury human waste. Because the outbreak was spreading rapidly and the initial case-fatality rate (CFR) was high, MSPP and the USG initially focused on 5 immediate priorities: 1) prevent deaths in health facilities by distributing treatment supplies and providing clinical training; 2) prevent deaths in communities by supplying oral rehydration solution (ORS) sachets to homes and urging ill persons to seek care quickly; 3) prevent disease spread by promoting point-of-use water treatment and safe storage in the home, handwashing, and proper sewage disposal; 4) conduct field investigations to define risk factors and guide prevention strategies; and 5) establish a national cholera surveillance system to monitor spread of disease.

### National Surveillance of Rapidly Spreading Epidemic

**Figure 1:**
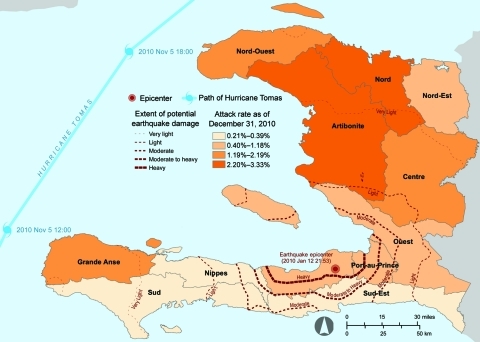
Administrative departments of Haiti affected by the earthquake of January 12, 2010; the path of Hurricane Tomas, November 5–6, 2010; and cumulative cholera incidence by department as of December 28, 2010.

**Figure 2:**
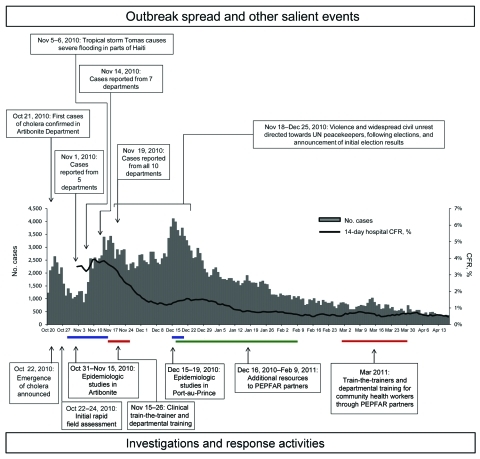
Reported cases of cholera by day, and 14-day smoothed case-fatality rate (CFR) among hospitalized cases, by day, Haiti, October 22, 2010–July 25, 2011. UN, United Nations; CDC, Centers for Disease Control and Prevention; PAHO, Pan American Health Organization; MSPP, Ministère de la Santé Publique et de la Population.

Health officials needed daily reports (which established reportable disease surveillance systems were not able to provide) to monitor the epidemic spread and to position cholera prevention and treatment resources across the country. In the first week of the outbreak, MSPP’s director general collected daily reports by telephone from health facilities and reported results to the press. On November 1, formal national cholera surveillance began, and MSPP began posting reports on its website (www.mspp.gouv.ht). On November 5–6, Hurricane Tomas further complicated surveillance and response efforts, and many persons fled flood-prone areas. By November 19, cholera was laboratory confirmed in all 10 administrative departments and Port-au-Prince, as well as in the Dominican Republic and Florida (*24*,*25*) (Figure 1). Though recently affected departments in Haiti experienced high initial CFRs, by mid December, the CFR for hospitalized case-patients was decreasing in most departments, and fell to 1% in Artibonite Department (*26*). Reported cases decreased substantially in January, and the national CFR of hospitalized case-patients fell below 1% (Figure 2). As of July 31, 2011, a total of 419,511 cases, 222,359 hospitalized case-patients, and 5,968 deaths had been reported.

### Field Investigations and Laboratory Studies

To guide the public health response, officials needed to know how cholera was being transmitted, which interventions were most effective, and how well the population was protecting itself. Therefore, CDC collaborated with MSPP and other partners to conduct rapid field investigations and laboratory studies. Central early findings included the following.

First, identifying untreated drinking water as the primary source for cholera reinforced the need to provide water purification tablets and to teach the population how to use them. Although most of the population had heard messages about treating their drinking water, many lacked the means to do so.

In addition, in Artibonite Department, those with cholera-like illness died at home, after reaching hospitals, and after discharge home, which suggests that persons were unaware of how quickly cholera kills and that the overwhelmed health care system needed more capacity and training to deliver lifesaving care. Also, water and seafood from the harbors at St. Marc and Port-au-Prince were contaminated with *V. cholerae*, which affirmed the need to cook food thoroughly and advise shipmasters to exchange ballast water at sea to avoid contaminating other harbors.

The epidemic strain was resistant to many antimicrobial agents but susceptible to azithromycin and doxycycline. Guidelines were rapidly disseminated to ensure effective antimicrobial drug treatment.

Cholera affected inmates at the national penitentiary in Port-au-Prince in early November, causing ≈100 cases and 12 deaths in the first 4 days. The problem abated after the institution’s drinking water was disinfected and inmates were given prophylactic doxycycline.

Finally, investigators found that epidemic *V. cholerae* isolates all shared the same molecular markers, which suggests that a point introduction had occurred. The epidemic strain differed from Latin American epidemic strains and closely resembled a strain that first emerged in Orissa, India, in 2007 and spread throughout southern Asia and parts of Africa (*27*). These hybrid Orissa strains have the biochemical features of an El Tor biotype but the toxin of a classical biotype; the later biotype causes more severe illness and produces more durable immunity (*28*,*29*). A representative isolate was placed in the American Type Culture Collection, and 3 gene sequences were placed in GenBank (*23*).

### Training Clinical Caregivers and Community Health Workers

CDC developed training materials (in French and Creole) on cholera treatment and on November 15–16 held a training-of-trainers workshop in Port-au-Prince for locally employed clinical training staff working at PEPFAR sites across all 10 departments. These materials were also posted on the CDC website (www.cdc.gov/haiticholera/traning). The training-of-trainers graduates subsequently led training sessions in their respective departments; 521 persons were trained by early December.

During the initial response ≈10,000 community health workers (CHWs), supported through the Haitian government and other organizations, staffed local first aid clinics, taught health education classes, and led prevention activities in their communities. Training materials for CHWs developed by CDC were distributed at departmental training sessions, shared with other nongovernmental organization (NGO) agencies, and used in a follow-up session for CHWs held on March 1–3, 2011 (see pages 2162–5). The CHW materials discussed treating drinking water by using several water disinfection products; how to triage persons coming to a primary clinic with diarrhea and vomiting; making and using ORS; and disinfecting homes, clothing, and cadavers with chlorine bleach solutions. Materials were posted on the CDC website as well.

### Working with Partners to Increase Capacity for Cholera Treatment

Supply logistics were daunting as cholera spread rapidly across Haiti. Sudden, unexpected surges in cases could easily deplete local stocks of intravenous rehydration fluids and ORS sachets, and resupplying them could be slow. The national supply chain, called Program on Essential Medicine and Supplies, was managed by MSPP, with technical assistance from the Pan American Health Organization, and received shipments of donated materials and distributed them to clinics.

Early in November the USG provided essential cholera treatment supplies through the US Agency for International Development’s Office of Foreign Disaster Assistance (OFDA) to the national warehouse and IDP camps. CDC staff also distributed limited supplies to places with acute needs. To complement efforts by MSPP and aid organizations to establish preventive and treatment services, OFDA provided emergency funding to NGO partners with clinical capacity.

When surveillance and modeling suggested that the spread of cholera across Haiti could outpace the public health response, the USG reached out to additional partners to expand cholera preventive services and treatment capacity. PEPFAR clinicians were authorized to assist with clinical management of cholera patients and participated in clinical training across the country. In December, CDC received additional USG emergency funds and awarded MSPP and 6 additional PEPFAR partners $14 million to further expand cholera treatment and prevention efforts through 4,000 CHWs and workers at 500 community oral rehydration points. Funds were also used to expand cholera treatment sites at 55 health facilities. In addition, CDC established the distribution of essential cholera supplies to PEPFAR partners through an existing HIV commodities supply chain management system.

### Improvements in Water, Sanitation, and Hygiene

To increase access to treated water and raise awareness of ways to prevent cholera, a consortium of involved NGOs and agencies, called the water, sanitation, and hygiene cluster, met weekly. Led by Haiti’s National Department of Drinking Water and Sanitation and the United Nation’s Children’s Fund, the members of this cluster targeted all piped water supplies for chlorination, and began distributing water purifying tablets for use in homes throughout Haiti. CDC helped the National Department of Drinking Water and Sanitation monitor these early efforts with qualitative and quantitative assessments of knowledge, attitudes, and practices. Emergency measures, especially enhanced chlorination of central water supplies, were expanded in the IDP camps because of the perceived high risk. OFDA and CDC provided water storage vessels, soap, and large quantities of emergency water treatment supplies for households and piped water systems. Distributing water purifying tablet supplies to difficult-to-reach locations remained a challenge.

### Educating the Public

**Figure 3:**
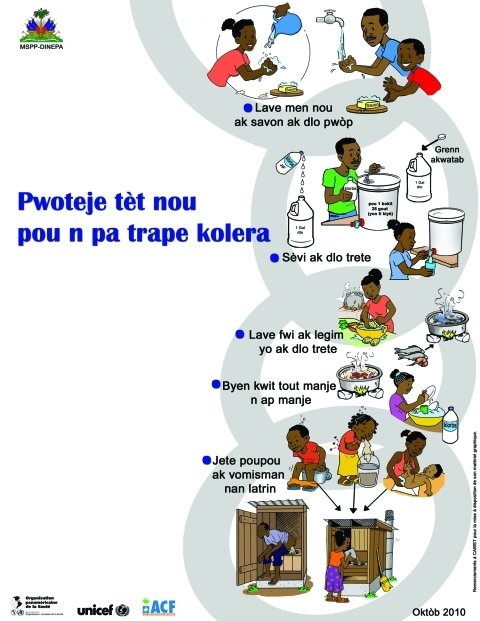
Educational poster (in Haitian Creole) used by the Haitian Ministère de la Santé Publique et de la Population (MSPP) to graphically present the ways of preventing cholera. DINEPA, Direction Nationale de l’Eau Potable et d’ Assainessement; UNICEF, United Nations Children’s Fund; ACF, Action Contre la Faim.

Beginning October 22, MSPP broadcast mass media messages, displayed banners, and sent text messages encouraging the population to boil drinking water and seek care quickly if they became ill. Early investigations affirmed the public’s need for 5 basic messages:1) drink only treated water; 2) cook food thoroughly (especially seafood); 3) wash hands; 4) seek care immediately for diarrheal illness; 4) and give ORS to anyone with diarrhea. In mid November, focus group studies in Artibonite indicated that residents were confused about how cholera was spreading and how to best prevent it, but they understood the need to treat diarrheal illness with ORS, how to prepare ORS, and how to disinfect water with water purification tablets (*30*). Posters provided graphic messages for those who could not read (Figure 3). On November 14, Haitian President René Préval led a 4-hour televised public conference to promote prevention, stressing home water treatment and handwashing, and comedian Tonton Bichat showed how to mix ORS.

## Cholera Epidemic in Dominican Republic

Compared with Haiti’s experience, the epidemic has been less severe in Dominican Republic. Though the countries share the island, conditions in Dominican Republic are better than in Haiti: the IMR is one third that of Haiti, gross domestic product per capita is 5× greater, and 86% of the population has access to improved sanitation. Within 48 hours of the report of cholera in Haiti, the Ministry of Health in the Dominican Republic and CDC established the capacity for diagnosing cholera at the national laboratory; the first cholera case was confirmed on October 31. Dominican officials quickly planned for cholera treatment centers in at least 70 hospitals, trained staff in primary care clinics and prison dispensaries, and stocked medical supplies sufficient to treat 20,000 cases. By December, 75% of doctors had received training in the management of cholera. Chlorination levels and water quality were monitored in municipal water systems across the country. The border with Haiti was not closed, and no major trade disruptions occurred. Sanitation improvements were instituted in border markets, schools, institutions, and mass gatherings. Public education in the first 3 months included dissemination of 4,300 mass media messages, nearly 3 million flyers, 50,000 classroom booklets for teachers, and a volunteer effort to visit 1 million homes. A survey of the knowledge, attitudes, and practices of residents of Santo Domingo showed that 89% had received cholera prevention messages. Transmission was limited, but sustained, in mid December and continued at low levels through the spring. One large outbreak affected guests at a wedding in January 2011, including some visitors from Venezuela and the United States (see pages 2172–4). From October 21, 2010, through July 30, 2011, a total of 14,598 suspected cases of cholera were reported; 256 persons died (of these, cases in 92 patients were laboratory confirmed) (*31*).

## Uncertainties and Challenges of Cholera in the Caribbean

Cholera may increase seasonally in Haiti each year (during the rainy season) as it did in 2011. The lack of a history of cholera in the Caribbean makes prediction a challenge because cholera seasonality varies from place to place. Other unknown factors are what proportion of the population has now been immunized by natural infection and how long this immunity might last. In a setting in which the population has poor access to clean water and sanitation, endemic transmission could persist for years if the epidemic strain finds long-term reservoirs in brackish coastal waters. Antimicrobial drug resistance may emerge in toxigenic *V. cholerae* O1, making continued monitoring of antimicrobial drug susceptibility essential.

Whether the epidemic will spread beyond Hispaniola is also uncertain. With the highest IMR in the Western Hemisphere (reflecting major gaps in sanitation and health care), Haiti is uniquely susceptible. Other countries in the Caribbean region have an IMR less than half that of Haiti (Guatemala is next with an IMR of 33), which suggests less risk for sustained transmission. If shipmasters leaving Haitian ports would exchange their ships’ ballast water at sea, they could help prevent the transfer of epidemic cholera from harbor to harbor.

The origin of cholera in Haiti also raises questions. It has been suggested that United Nations peacekeeping troops from Nepal may have introduced cholera into Haiti (*32*). Genetic comparison of the Haitian epidemic strain with other strains from around the world suggests that it resembles strains seen in southern Asia and African (*23*) and strains from Nepal (*33*). Although knowing how cholera was introduced into Haiti would not help dampen its spread throughout Hispaniola, the knowledge might help foster disease monitoring and sanitation policies that would prevent such introductions elsewhere (*34*).

A continuing challenge facing Haiti is how to manage cholera treatment with limited resources. Cholera training for doctors and nurses should be added to clinical curricula. By increasing use of ORS and expanding the antimicrobial drug treatment of hospitalized patients, intravenous fluid needs might be decreased, without posing an undue risk for antimicrobial drug resistance. Focusing on supply chain logistics is critical to ensuring the maintenence of tenuous buffer stocks of supplies.

Residents of IDP camps have been largely spared from the outbreak because of safer water supplies and improved sanitation in the camps, but preserving that protection as persons move on to homes without piped water or sewage systems will be a challenge. Encouraging and empowering residents to disinfect drinking water in their homes, schools, and clinics by using chlorine products has been effective in many African and Latin American countries and is a practical interim solution for Haiti (*35*).

The role of oral cholera vaccine in the immediate postepidemic period continues to be evaluated (*36*,*37*). Both the global cholera vaccine supply and Haitian vaccine cold chain are currently insufficient to mount national vaccination campaigns on Hispaniola. A limited vaccination pilot study could increase our global understanding of the costs, benefits, and practical applicability of using oral cholera vaccine in such circumstances.

## Lessons Learned

The existing PEPFAR program that provided support for clinical care delivery and public health infrastructure was a powerful framework that sustained the national cholera response in Haiti. Through additional USG funding for PEPFAR partners, an expanded cadre of Haitian clinicians and CHWs received cholera training, resulting in expanded access to cholera treatment throughout Haiti. In addition, the postearthquake enhancement of diagnostic laboratory testing capacity for reportable diseases enabled health officials to quickly confirm the cholera outbreak and monitor antimicrobial drug susceptibility of the bacterial strains.

The Haitian epidemic shows that as long as cholera exists anywhere in the world, many who drink untreated water and live in areas of poor sanitation are at risk. The epidemic also shows how cholera can emerge where it is least expected. Despite heightened efforts to detect acute watery diarrhea among persons in urban IDP camps, cholera appeared first in rural Haiti, just as in Mexico in the 1990s, where it first emerged unexpectedly in a remote mountainous region (*8*). Therefore, the ability to detect and confirm cholera needs to be broadly available.

The Haitian experience also shows the continued success of the rehydration treatment strategies first developed in Bangladesh and refined over the past 40 years. With training and adequate supplies and treatment facilities, hospitalized case-fatality ratios of <1% were achieved. If the improvements in ORS use in treatment of diarrheal illness are sustained, these actions could reduce childhood deaths permanently.

The more moderate course of the epidemic in the Dominican Republic and the relative sparing of the IDP camps in Haiti illustrate how safer water and better sanitation can prevent transmission. Without these basic public health bulwarks, the risk for recurrent cholera and other major waterborne diseases remains high. In the interim, safe water and handwashing practices should be integrated into household and community settings (*35*).

## Investing in Safe Water and Sanitation

Global experience with cholera suggests that the epidemic in Haiti could last for years. Although case counts decreased in early 2011, cases again increased with the onset of the rainy season, and conditions that permit waterborne transmission persist. Improving Haiti’s water and sanitation infrastructure is critical to achieving the same profound health gains brought by improved water and sanitation infrastructure elsewhere (*3*,*6*,*38*).

The World Health Organization estimates that meeting the global Millennium Development Goal for improving access to safe water and improved sanitation would have a huge return on investment worldwide (*39*). For each $1 invested, the economic rate of return in regained time at work and school, time saved at home by not hauling water, increased productivity, and reduced health costs would be as much as $8, in addition to the direct health benefits. For Haiti to meet this goal, an estimated 250,000 households would need access to an improved water source, and ≈1 million families would need access to improved sanitation. The Inter-American Development Bank estimated in 2008 that Haiti would require $750 million to achieve this goal (*40*). After the earthquake, the international community pledged >$6 billion to Haiti for relief. A long-term plan to build safe drinking water and sewerage systems is well within the range of the resources pledged.
