# Correction: Vol. 17, No. 11

**DOI:** 10.3201/eid1712.C11712

**Published:** 2011-12

**Keywords:** Correction: Vol. 17, No. 11, Erratum, Tappero, 11-0827

In the article Lessons Learned during Public Health Response to Cholera Epidemic in Haiti and the Dominican Republic (J.W. Tappero, R.V. Tauxe), data in Figure 2 were published incorrectly. The article has also been corrected online (http://wwwnc.cdc.gov/eid/article/17/11/c1-1712_article.htm).

